# Impact of Plastic-Wrap Properties and Cleaning Intervals on the Disinfection of Elevator Buttons

**DOI:** 10.3390/ijerph20021649

**Published:** 2023-01-16

**Authors:** Shin-Huei Kuo, Tzu-Yin Liu, Tun-Chieh Chen, Chih-Jen Yang, Yen-Hsu Chen

**Affiliations:** 1 Department of Internal Medicine, Kaohsiung Municipal Ta-Tung Hospital, Kaohsiung Medical University, No. 68, Jhonghua 3rd Road, Kaohsiung 80145, Taiwan; 2 Division of Infectious Diseases, Department of Internal Medicine, Kaohsiung Medical University Hospital, Kaohsiung Medical University, No. 100, Shih-Chuan 1st Road, Kaohsiung 80708, Taiwan; 3 Infection Control Office, Kaohsiung Municipal Ta-Tung Hospital, Kaohsiung Medical University, No. 68, Jhonghua 3rd Road, Kaohsiung 80145, Taiwan; 4 School of Medicine, College of Medicine, Kaohsiung Medical University, No. 100, Shih-Chuan 1st Road, Kaohsiung 80708, Taiwan; 5 Division of Pulmonary and Critical Care Medicine, Department of Internal Medicine, Kaohsiung Medical University Hospital, Kaohsiung Medical University, No. 100, Shih-Chuan 1st Road, Kaohsiung 80708, Taiwan; 6 School of Medicine, College of Medicine, National Sun Yat-sen University, No. 70, Lien-Hai Road, Kaohsiung 80424, Taiwan

**Keywords:** elevator buttons, plastic wraps, adenosine triphosphate bioluminescence assay, disinfection, cleaning interval

## Abstract

Fomite transmission is a possible route by which different pathogens spread within facilities. In hospital settings, elevator buttons are widely observed to be covered with various types of plastic wraps; however, limited information is available concerning the impact of different plastic materials on cleaning. Our study aimed to identify which plastic material is suitable for the coverage of elevator buttons and the optimal intervals for their cleaning. We tested six plastic covers, including polyethylene (PE), polymethylpentene (PMP), polyvinyl chloride (PVD), and polyvinylidene chloride (PVDC) plastic wraps; a thermoplastic polyurethane (TPU) keyboard cover; and a polyethylene terephthalate-ethylene vinyl acetate (PET-EVA) laminating film, which are plastic films. The bioburden on the elevator buttons at different time intervals was measured using an adenosine triphosphate (ATP) bioluminescence assay. Our results show that wraps made of PVDC had superior durability compared with those of PMP, PVC, and PVDC, in addition to the lowest detectable ATP levels among the six tested materials. Regarding different button locations, the highest ATP values were found in door-close buttons followed by door-open, and first-floor buttons after one- and three-hour intervals (*p* = 0.024 and *p* < 0.001, respectively). After routine disinfection, the ATP levels of buttons rapidly increased after touching and became more prominent after three hours (*p* < 0.05). Our results indicate that PVDC plastic wraps have adequate durability and the lowest residual bioburden when applied as covers for elevator buttons. Door-close and -open buttons were the most frequently touched sites, requiring more accurate and precise disinfection; therefore, cleaning intervals of no longer than three hours may be warranted.

## 1. Introduction

Direct contact with infected or colonized patients and objects leads to transmission that may result in considerable hospital-acquired infection (HAI) [1,2,3]. Surfaces represent a meaningful facilitator of disease transmission because they can act as a reservoir of microorganisms that may spread to whoever comes in contact with them [4,5,6]. Gram-positive and -negative bacteria can survive for months on dry, inanimate surfaces in hospitals, and severe acute respiratory syndrome coronavirus 2 (SARS-CoV-2) is shown to be detectable for up to seven days on plastic surfaces [7,8,9].

Elevators are ubiquitous and active inside hospitals, and their buttons are under-recognized sites of microbial contamination [10,11]. Because of concerns regarding damage to elevator buttons resulting from frequent bleaching in communities and hospitals, they are commonly covered by plastic wraps or films [12]. Plastic wraps have excellent adhesion, low cost, and lightweight properties compared with a series of composite biocides commercially available for “self-disinfecting surfaces” [13,14,15]. However, there is little available information about the effects of various plastic materials on disinfection.

Moreover, updated guidelines or reviews always highlight the importance of cleaning and disinfecting surfaces in hospitals [16,17,18]. Globally, one of the most popular methods to evaluate the quality of cleaning is the adenosine triphosphate (ATP) bioluminescence assay, which provides rapid feedback on bacterial load and bioburden monitoring [19,20]. Nevertheless, prior to our study, there has been a lack of concise recommendations for elevator cleaning intervals, which are not covered even in the Asia Pacific Society of Infection Control (APSIC) guidelines [21].

In our study, we aimed to evaluate the impact of different plastic materials on elevator button coverage, identify the most frequently contaminated sites on the panel, and determine an adequate interval for cleanliness based on our ATP method results.

## 2. Materials and Methods

### 2.1. Study Design

For this study, we conducted a comparative analysis of samples from the four main elevators in a 395-bed regional hospital. Each elevator had eleven buttons, including door-close, door-open, basement 1, and floors 1–9. The third floor contained outpatient and traditional Chinese medicine services. In general practice, the elevator panels were cleaned three times per day without fixed intervals. We tested the uncovered panel (null) and six different materials, including four plastic wraps (polyethylene (PE), polymethylpentene (PMP), polyvinyl chloride (PVC), and polyvinylidene chloride (PVDC)) and two plastic films, polyethylene terephthalate-ethylene vinyl acetate (PET-EVA) laminating films and thermoplastic polyurethane (TPU) keyboard covers (products for keyboards but being applied to the elevator button board) (Rt-SC03; ROTA America Inc., San Jose, CA, USA) (Figure S1). We applied plastic wraps or films to the elevator panels and performed routine disinfection using 500 ppm sodium hypochlorite (6% bleach; Jenn Feng Chemical Works Co., Ltd., New Taipei City, Taiwan). The ATP values were determined for elevator buttons sampled between April 2020 and May 2020.

### 2.2. Investigation and Sampling Process

First, we investigated the condition of the four wraps on elevator panels at three different time points (ten minutes, one hour, and three hours) and defined them as intact or ruptured. Second, we collected a background ATP value when applying a new plastic wrap or film after cleaning them with 75% ethanol. After completing routine cleaning and disinfection, all elevator buttons in each forum were assessed by swabbing the entire surface of each button (10.5 cm^2^) in one direction and then in the opposite direction. Simultaneously, the ATP values of other residual, non-frequently touched areas on the elevator panel were assessed for comparison. Except for a swab for the ATP assay (3M Clean-Trace System; 3M, St. Paul, MN, USA), a premoistened sterile culture swab for the aerobic colony count (ACC) method (BBL CultureSwab; Becton Dickinson, Franklin Lakes, NJ, USA) was applied. The ATP assay was used to evaluate the cumulative bioburden of surfaces by first activating the swabs following the manufacturer’s instructions and then recording the reading (in relative light units, RLUs). Each culture swab was suspended in 1 mL sterile saline, then vortexed for 10 s, and 0.2 mL was spread onto a tryptic soy agar (Creative Microbiologicals, Taipei County, Taiwan). After 48 h of incubation at 35 °C, the total number of colonies on the agar was calculated for all samples. Nevertheless, we used the ACC method only for the first time while investigating six different plastic materials and the uncovered panel (null). The same well-trained infection control nurse conducted all tests. No further cleaning or disinfection processes were implemented whilst obtaining the serial ATP values from the same tested materials (Figure S2). Each day, tested plastic covers were replaced with new ones before initiating a new investigation.

### 2.3. Statistical Analysis

Data were recorded in an Excel database (Microsoft, Redmond, WA, USA) and analyzed using IBM SPSS Statistics (Version 20.0; Armonk, NY, USA: IBM Corp.). The ATP values are expressed as the mean and standard deviation (SD). Pairwise differences were assessed using one-way analysis of variance (ANOVA) with Student–Newman–Keuls post hoc tests. Results with two-sided *p* values less than 0.05 were considered to indicate statistically significant differences.

## 3. Results

### 3.1. Comparison of PE, PMP, PVC, and PVDC Wraps

In total, 24 observation events were recorded for each plastic wrap at ten minutes, one hour, and three hours after bleach cleaning. PVDC (100%, 24/24) had the best durability compared with PE (66.7%, 16/24), PMP (54.2%, 13/24), and PVC (54.2%, 13/24).

The summation of ATP values from all elevators at different dates was similar (*p* = 0.409). After pooling ATP values from all buttons after one hour of use, PVDC was found to have lower values (mean ± standard deviation (SD), 147.3 ± 263.6 RLU) than PE (220.5 ± 246.5 RLU), PVC (252.5 ± 278.3 RLU), and PMP (276.6 ± 222.8 RLU) although the difference was not statistically significant (*p* = 0.491). Moreover, pooled ATP values from other residual areas (excluding eleven buttons) were relatively lower except at the three-hour time point (range: 19–77 RLU), no matter which plastic wrap was used. Regarding the different button locations, we detected the highest ATP values at the one-hour interval on door-close buttons (378.5 ± 301.7 RLU), followed by door-open (281.5 ± 282.9 RLU) and first-floor buttons (167.5 ± 146.5 RLU) (*p* < 0.001, ANOVA).

A consistent trend was found in the ACC method. PVDC had the lowest pooled ACC values from all buttons after one hour of use (15 colony forming units (CFU)/10.5 cm^2^), followed by PE (32 CFU/10.5 cm^2^), PVC (42 CFU/10.5 cm^2^), and PMP (58 CFU/10.5 cm^2^). At the three-hour time point, the trend remained unchanged (PVDC: 28 CFU/10.5 cm^2^, PE: 57 CFU/10.5 cm^2^, PVC: 80 CFU/10.5 cm^2^, and PMP: 115 CFU/10.5 cm^2^). In addition, the ACC values of other residual areas (excluding eleven buttons) were extremely low except at the three-hour time point (range: 1–5 CFU), no matter which plastic wrap was used. The highest ACC value was detected on door-close buttons either after one-hour intervals (PVDC: 4 CFU/10.5 cm^2^, PE: 14 CFU/10.5 cm^2^, PVC: 24 CFU/10.5 cm^2^, and PMP: 26 CFU/10.5 cm^2^) or three-hour intervals (PVDC: 16 CFU/10.5 cm^2^, PE: 23 CFU/10.5 cm^2^, PVC: 32 CFU/10.5 cm^2^, and PMP: 64 CFU/10.5 cm^2^).

### 3.2. Comparison of TPU Keyboard Cover, PET-EVA Laminating Film, PVDC Wrap, and Uncovered Panel (Null)

**Figure 1:**
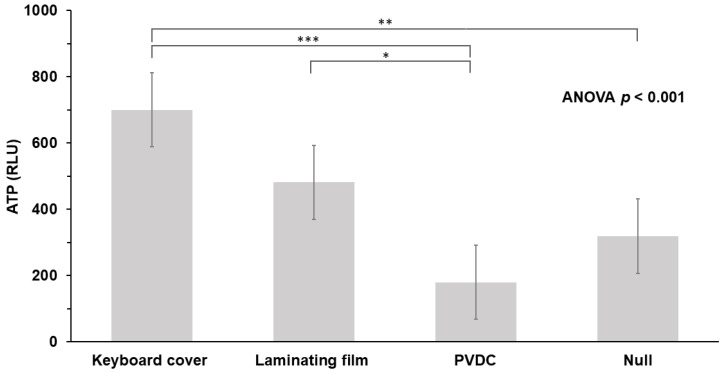
Values of ATP on two different plastic films, PVDC wrap and uncovered panel (null). ATP, adenosine triphosphate; RLU, relative light unit; PVDC, polyvinylidene chloride; * *p* < 0.05, ** *p* < 0.01, *** *p* < 0.001.

All of our tested TPU keyboard covers, PET-EVA laminating films, and PVDC wraps remained intact throughout the study. The summated ATP values for all elevators at different dates from different time intervals during the study period were not found to be statistically different (one hour, *p* = 0.151; three hours, *p* = 0.506). After one hour of use, the PVDC wraps had significantly lower ATP values (180.0 ± 122.1 RLU) than TPU keyboard covers (700.0 ± 553.6 RLU), PET-EVA laminating films (482.0 ± 275.7 RLU), and uncovered panel (null) (319.1 ± 205.7 RLU) (*p* < 0.001, ANOVA) (Table 1, Figure 1). The PVDC wraps remained with the lowest ATP values at three hours, although the finding is not statistically significant (*p* = 0.073) (Table 1). However, summated ATP values from other residual areas (excluding eleven buttons) were relatively lower despite at the three-hour time point (range: 19–162 RLU) no matter which material was used or the uncovered panel (null).

**Figure 2:**
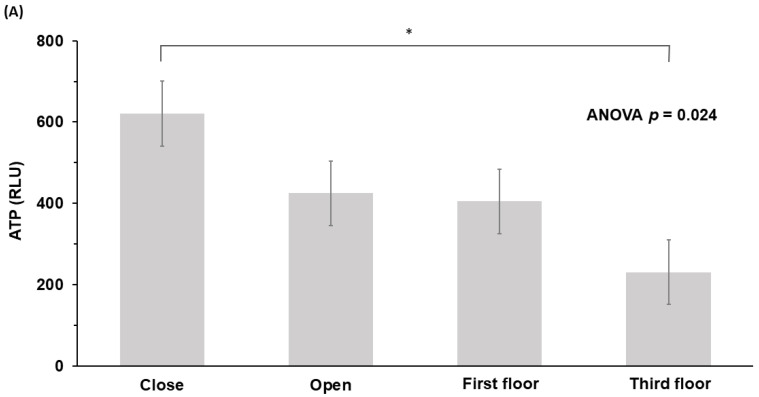

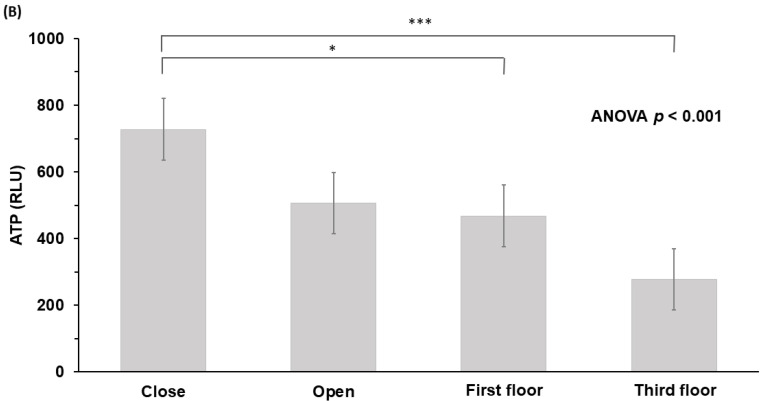
Values of ATP on different button locations at (**A**) one-hour intervals and (**B**) three-hour intervals. ATP, adenosine triphosphate; RLU, relative light unit; * *p* < 0.05, *** *p* < 0.001.

ATP values measured at one-hour intervals were highest for door-close buttons (620.7 ± 489.3 RLU), followed by door-open buttons (424.8 ± 223.7 RLU), first-floor (404.8 ± 387.5 RLU), and third-floor buttons (230.7 ± 277.6 RLU) (*p* = 0.024, ANOVA) (Table 2, Figure 2A). At the three-hour time point, our results were: door-close (727.4 ± 482.3 RLU), door-open (505.9 ± 262.9 RLU), first-floor (468.1 ± 356.8 RLU), and third-floor buttons (277.8 ± 288.4 RLU) (*p* < 0.001, ANOVA) (Table 2, Figure 2B).

A consistent trend was found in the ACC method. PVDC had the lowest pooled ACC values from all buttons after one hour of use (15 CFU/10.5 cm^2^), followed by uncovered panels (null) (22 CFU/10.5 cm^2^), PET-EVA laminating films (32 CFU/10.5 cm^2^), and TPU keyboard covers (52 CFU/10.5 cm^2^). At the three-hour time point, the trend remained unchanged (PVDC: 28 CFU/10.5 cm^2^, uncovered panels (null): 34 CFU/10.5 cm^2^, PET-EVA laminating films: 41 CFU/10.5 cm^2^, and TPU keyboard covers: 82 CFU/10.5 cm^2^). In addition, the ACC values of other residual areas (excluding eleven buttons) were relatively lower except at the three-hour time point (range: 1–14 CFU), no matter which material was used or the uncovered panel (null). The highest ACC value was detected on door-close buttons either after one-hour intervals (PVDC: 4 CFU/10.5 cm^2^, uncovered panels (null): 14 CFU/10.5 cm^2^, PET-EVA laminating films: 16 CFU/10.5 cm^2^, and TPU keyboard covers: 22 CFU/10.5 cm^2^) or three-hour intervals (PVDC: 16 CFU/10.5 cm^2^, uncovered panels (null): 21 CFU/10.5 cm^2^, PET-EVA laminating films: 24 CFU/10.5 cm^2^, and TPU keyboard covers: 38 CFU/10.5 cm^2^).

**Figure 3:**
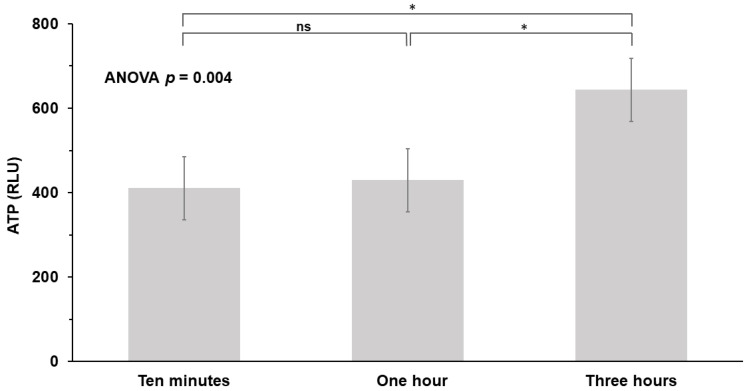
Values of ATP at different time points: ten minutes, one hour, and three hours. ATP, adenosine triphosphate; RLU, relative light unit; ns, no significance; * *p* < 0.05.

Pooled ATP values from buttons at different time intervals, including ten minutes (410.8 ± 410.3 RLU), one hour (429.7 ± 351.1 RLU), and three hours (644.0 ± 363.4 RLU), were significantly different (*p* = 0.004, ANOVA) (Figure 3).

## 4. Discussion

Our findings indicate that the PVDC wrap had the best durability and the lowest ATP values compared with PE, PVC, and PMP after usage as elevator button covers. Moreover, a cleaning interval of fewer than three hours was reasonable for the most frequently touched sites, such as door-close and -open buttons.

In our study, we chose plastic wraps and films commonly encountered in ordinary life because they are an economical option in low- and middle-income countries [22]. PVDC wraps showed considerable durability and the lowest detectable ATP values after usage. According to data from previous studies, PVDC offers considerable durability, very low moisture regain, and is also impervious to mold formation, bacteria, and insect damage, thereby accounting for its lowest detectable bioburden of those tested [23,24]. By contrast, PET/EVA laminating films have insulating properties and are therefore susceptible to electrostatic charging, and TPU keyboard covers have undetectable surface roughness owing to different exposure processes, which might explain their unexpectedly high ATP values in our study [25,26]. Surface modification with anti-adhesive properties, incorporating antimicrobial substances, or modification with biologically active metals are some of the recently proposed strategies for overcoming surface contamination problems [13,14,15,27]. Although most of the results are promising, the associated costs of the strategies are high, and the possibility of antimicrobial resistance during declined efficacy requires further exploration.

Numerous studies have established the hospital environment as responsible for transmitting critical nosocomial pathogens to patients [4,5,6]. In the past decade, studies have also demonstrated the role of “non-classical” surfaces, such as stethoscopes, computer keyboards, elevator buttons, and mobile communication devices, in pathogen transmission via contact [4,10,21,28,29,30]. Our study focused on frequently touched elevator panels and confirmed that the door-close buttons were the most contaminated sites, followed by the door-open buttons. Although our results could not specify whether there is a risk of SARS-CoV-2 transmission, a cross-sectional study that traced SARS-CoV-2-infected patients and their close contacts and concluded infections occurred through touching of contaminated elevator buttons, supposed elevators are one fomite situation that we cannot afford to ignore [31]. Moreover, Bhatta et al. investigated the diversity and distribution of bacterial contamination from frequently touched surfaces shared by healthcare workers, patients, and visitors and reported that large quantities of *Staphylococcus aureus* isolates were recovered from elevator buttons (25%), which was second only to door handles (29.5%) [10]. Furthermore, there is a possible correlation with poor personal hygiene and ineffective cleaning, sanitizing, and disinfecting routine schedules.

Under the traffic control bundle, our data showed that an appropriate interval for the effective cleaning of frequent-touched elevator buttons was less than three hours due to ATP levels rising rapidly after touching [32]. This finding might contribute to further comprehensive, multicomponent environmental cleaning and disinfection intervention. However, the optimal cleaning interval for nonpatient-care areas remains unresolved due to a lack of prospective comparison studies and the ecological complexity of healthcare settings [17,21]. In addition, there is a wide variety of biological material that is measured by using ATP, of which the bacterial load is only one of many components. The contamination of surfaces with organic materials could provide a nutritional source for microbial pathogens, and Lee et al. demonstrated that a decrease in microbial growth, contributing to a clean hospital environment, can reduce the risk of microbial transmission [33]. Nevertheless, the correlation between the amount of ATP measured and microbial contamination within the healthcare setting was not well documented, and various studies reported different correlations [34,35,36,37]. Another point of discussion is the correlation between the amount of ATP measured and the expressed RLU. Omidbakhsh et al. conclude that there is a strong positive correlation between true concentrations of ATP and RLU readings; however, this correlation is best only when the concentration of ATP is higher [38]. Apart from its limited sensitivity in detecting low levels of microbial contamination, the ATP meters tested were also prone to interference by different disinfectant chemistries [38]. Environmental factors in healthcare settings, including temperature, humidity, stability of fomites, and ventilation and filtering systems, could significantly influence pathogen propagation [17,39]. Adequate control of these environmental factors and hand hygiene will strengthen infection control and prevent unnecessary healthcare-associated infections [16].

Our study has several limitations. First, we could not make a direct head-to-head comparison of these six tested materials as only four main elevators were available. Second, hand hygiene habits, crowds of people, temperature, and humidity all contributed to elevator button conditions. Although different data may be obtained during different study periods, we expect that the trend will remain unchanged. Third, ATP measurements are correlated with organic matter from microbial origins and blood, protein tissues, and epithelial cells [35,40]. Because there was no COVID-19 outbreak in Taiwan from April to May 2020, we did not perform a real-time reverse-transcription polymerase chain reaction analysis for SARS-CoV-2 via environmental sampling to evaluate the association between viral contamination and ATP measurement.

## 5. Conclusions

Our investigation results support the application of PVDC wrap in elevator button coverage because of its fair durability and lower accumulation of bioburdens compared with other tested plastic materials. Door-close and door-open buttons are the most frequently touched sites on the elevator panel; therefore, cleaning intervals of no longer than three hours may be warranted for effective disinfection.

## Figures and Tables

**Table 1:** Comparison of ATP values on different plastic materials and uncovered panel (null) (*n* = 144).

ATP (RLU)	TPU Keyboard Cover (*n* = 36) (a) Mean ± SD	PET-EVA Laminating Film (*n* = 36) (b)	PVDC Wrap (*n* = 36) (c)	Null (*n* = 36) (d)	*p*	Post Hoc ^a^
One hour	700.0 ± 553.6	482.0 ± 275.7	180.0 ± 122.1	319.1 ± 205.7	<0.001	a > c ***, a > d **, b > c *
Three hours	726.7 ± 514.1	522.6 ± 320.0	265.8 ± 208.3	464.2 ± 306.4	0.073	

ATP, adenosine triphosphate; RLU, relative light unit; TPU, thermoplastic polyurethane; PET-EVA, polyethylene terephthalate-ethylene vinyl acetate; PVDC, polyvinylidene chloride; SD, standard deviation. ^a^ * *p* < 0.05, ** *p* < 0.01, *** *p* < 0.001.

**Table 2:** Comparison of ATP values on buttons of different locations (*n* = 144).

ATP (RLU)	Door Close (*n* = 36) (a) Mean ± SD	Door Open (*n* = 36) (b)	First Floor (*n* = 36) (c)	Third Floor (*n* = 36) (d)	*p*	Post Hoc ^a^
One hour	620.7 ± 489.3	424.8 ± 223.7	404.8 ± 387.5	230.7 ± 277.6	0.024	a > d *
Three hours	727.4 ± 482.3	505.9 ± 262.9	468.1 ± 356.8	277.8 ± 284.4	<0.001	a > c *, a > d ***

ATP, adenosine triphosphate; RLU, relative light unit; SD, standard deviation. ^a^ * *p* < 0.05, *** *p* < 0.001.

## Data Availability

The data presented in this study are available on request from the corresponding author.

## Supplementary Materials

The following supporting information can be downloaded at: https://www.mdpi.com/article/10.3390/ijerph20021649/s1. Figure S1. The thin, stretchy, and durable TPU keyboard covers were used for (A) keyboard protection and (B) elevator button board coverage (in the current study). Figure S2. Timing of sampling while initiating an investigation of each plastic cover.
