# Supplementary material for: Impact of Plastic-Wrap Properties and Cleaning Intervals on the Disinfection of Elevator Buttons
Source: Int J Environ Res Public Health. 2023 Jan 16;20(2):1649. doi: 10.3390/ijerph20021649 (PMC9863425; doi:10.3390/ijerph20021649)
Supplement: Supplementary file 1 [file ijerph-20-01649-s001.zip › ijerph-2001857-supplementary.docx]

**Supplementary Materials:**


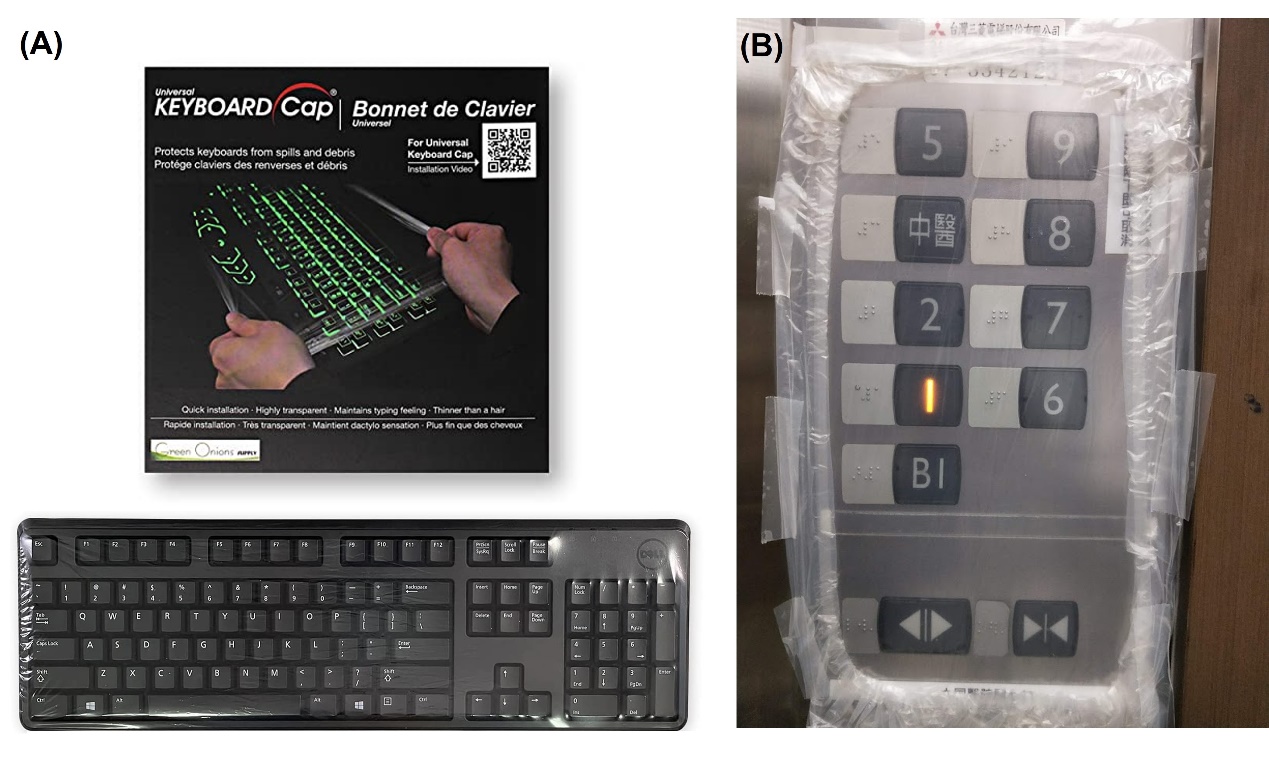


**Figure S1.** The thin, stretchy, and durable TPU keyboard covers were used for (**A**) keyboard protection and (**B**) elevator button board coverage (in the current study). TPU, thermoplastic polyurethane.


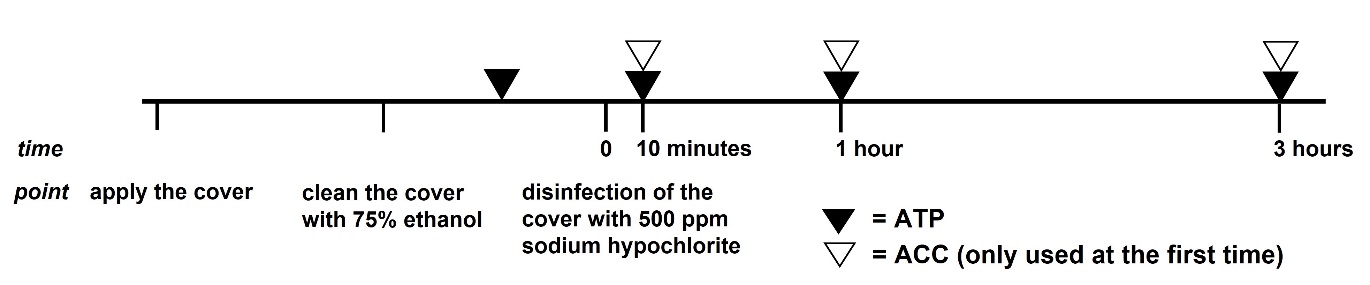


**Figure S2.** Timing of sampling while initiating an investigation of each plastic cover. ATP, adenosine triphosphate; ACC, aerobic colony count.
